# No Evidence of Dehydration with Moderate Daily Coffee Intake: A Counterbalanced Cross-Over Study in a Free-Living Population

**DOI:** 10.1371/journal.pone.0084154

**Published:** 2014-01-09

**Authors:** Sophie C. Killer, Andrew K. Blannin, Asker E. Jeukendrup

**Affiliations:** School of Sport and Exercise Sciences, University of Birmingham, Birmingham, West Midlands, United Kingdom; University of Bath, United Kingdom

## Abstract

It is often suggested that coffee causes dehydration and its consumption should be avoided or significantly reduced to maintain fluid balance. The aim of this study was to directly compare the effects of coffee consumption against water ingestion across a range of validated hydration assessment techniques. In a counterbalanced cross-over design, 50 male coffee drinkers (habitually consuming 3–6 cups per day) participated in two trials, each lasting three consecutive days. In addition to controlled physical activity, food and fluid intake, participants consumed either 4×200 mL of coffee containing 4 mg/kg caffeine (C) or water (W). Total body water (TBW) was calculated pre- and post-trial via ingestion of Deuterium Oxide. Urinary and haematological hydration markers were recorded daily in addition to nude body mass measurement (BM). Plasma was analysed for caffeine to confirm compliance. There were no significant changes in TBW from beginning to end of either trial and no differences between trials (51.5±1.4 vs. 51.4±1.3 kg, for C and W, respectively). No differences were observed between trials across any haematological markers or in 24 h urine volume (2409±660 vs. 2428±669 mL, for C and W, respectively), USG, osmolality or creatinine. Mean urinary Na^+^ excretion was higher in C than W (p = 0.02). No significant differences in BM were found between conditions, although a small progressive daily fall was observed within both trials (0.4±0.5 kg; p<0.05). Our data show that there were no significant differences across a wide range of haematological and urinary markers of hydration status between trials. These data suggest that coffee, when consumed in moderation by caffeine habituated males provides similar hydrating qualities to water.

## Introduction

Maintenance of fluid balance is essential to sustain human life. Water intake balances fluid losses to achieve adequate hydration of bodily tissues. Although there are widespread guidelines in scientific literature and media for achieving optimal hydration status and about the effects that various caffeinated beverages may have on fluid balance, there is no clear consensus about how much fluid an individual should consume [1]. One study found total daily fluid intake observed in healthy adults varied from 0.416–4.316 L/day [2]. The current EFSA dietary references values for water intakes for male adults is 2.5 L/day [3]. However, published guidelines range from 1.5 L/day [4] to 3.7 L/day [5] for adult males. It has been suggested that caffeinated beverages should not be included in daily fluid requirement guidelines [6] and that a glass of water should be consumed with every cup of coffee or tea to ensure hydration is maintained [7].

Caffeine (1, 3, 7-trimethylxanthine) is a naturally occurring methylxanthine which can be found in coffee, tea and chocolate. Caffeine acts as an adenosine receptor antagonist to reduce fractional sodium reabsorption in both the proximal tubule and distal nephron. When consumed in large doses (≥500 mg), caffeine elicits a diuretic effect [8]–[10]. The diuretic potential of caffeine in humans has been researched for many years, with the first scientific report published over 80 y ago [11]. The authors of these early findings suggested that whilst caffeine causes acute diuresis, regular caffeine consumption may lead to a tolerance developing against its diuretic effect. It has since been suggested that caffeine withdrawal of as little as 4 days is sufficient for tolerance to be lost [12]. Following the work of Eddy & Downs [11], there has been a range of studies that have investigated the effects of caffeine on hydration status (Table 1). These studies report observations across a range of caffeine forms and doses on various markers of hydration status in either caffeine-habituated or caffeine-naive populations (individuals who do not habitually consume caffeine, or those who have abstained from caffeine consumption for ≥4 days). Although the data are somewhat varied, the general trend is that higher doses of caffeine in caffeine-naive individuals will elicit an acute increase in urine volume, yet a low to moderate dose of caffeine does not induce a diuretic effect [13]–[17].

**Table 1:** Effect of caffeine consumption on urine production.

Authors	Caffeine Dose *(mg)*	Caffeine Source	Test population	Diuretic Effect
[8]	4 mg/d (200–700 mg)	Caffeine tablet	Habituated caffeine users	Yes –during first day only
[9]	490–680 (8.7 mg/kg bw)	Caffeine powder added to carbohydrate electrolyte drink Vs carbohydrate electrolyte drink	Caffeine naïve (Habitual coffee drinkers – 4 day pre-trial deprivation)	Yes
[10]	642	Caffeinated coffee	Caffeine naïve (Habitual coffee drinkers – 5 day pre-trial deprivation)	Yes
[14]	452	Caffeine tablet	Habitual caffeine users – 98±17 mg/day	No
[17]	360	Caffeine tablet	NA	Yes
[15]	300	Caffeine tablet	Habitual coffee drinkers – 8 h pre-trial deprivation	No
[25]	250	Caffeine beverage	Caffeine naïve (Non-coffee drinkers – 3 week caffeine deprived)	Yes
[31]	250	Caffeine tablet	Caffeine naïve (1 week caffeine deprived)	Yes –during first hour only
[32]	240	Caffeine beverage	Caffeine habituated users	Yes
[14]	226	Caffeine capsules	Habitual caffeine users – 98±17 mg/day	No
[16]	168 and 252	Caffeinated tea	Caffeine users – 12 h deprived	No
[13]	114–253	Caffeinated carbonated cola, Caffeinated carbonated, non-caloric cola, Instant coffee	Caffeine habituated (61–464 mg/day)	No
[17]	45, 90 and 180	Caffeine tablet	NA	No

Coffee is comprised of many bioactive compounds in addition to caffeine. These active compounds may interact with each other and therefore coffee consumption cannot be directly compared to caffeine consumption in its purest form (1,3,7-trimethyl xanthine) [18]. Interestingly, only two studies have specifically investigated the effects of caffeine in the form of coffee on hydration status. One study investigated the effects of six cups of coffee (624 mg caffeine) on urine excretion following a five day caffeine deprivation period [10]. Over the 24 h period, authors report a 2.7% decrease in total body water and a 41% increase in urine excretion, with a subsequent 66% and 28% increase in urinary sodium and potassium excretion, respectively. Due to the study design, which only included caffeine habituated participants who abstained from caffeine for 5 days prior to testing, the results of the study should be interpreted with caution before applying to habitual moderate-intake coffee drinkers. Another study investigated the effects of consuming equal amounts of water, caffeinated cola and caffeinated coffee (3.1±0.4 mg/kg caffeine/day) against water with a mixture of caffeinated colas (1.4±0.2 mg/kg caffeine/day) or non-caffeinated beverages [13]. The authors found no effects of coffee consumption compared with non-caffeinated beverages across a range of hydration markers in caffeine-habituated participants. Whilst the authors concluded that the advice to exclude caffeinated beverages from daily fluid requirement was not supported by their findings, the study did not measure total body water. Total body water (TBW) estimations using the doubly labelled water dilution technique is considered the gold standard method for assessing body water fluctuations over time [14]. One recent study investigated the effects of caffeine on TBW using deuterium oxide [19]. Thirty participants classified as low caffeine users (<100 mg/day) consumed caffeine (5 mg/kg/day) or placebo tablets for 4 consecutive days. Although these participants could not be classified as ‘caffeine-habituated’, no changes in TBW measured on day 1 and 4 were found between the caffeine or placebo group. The authors suggested that a moderate dose of caffeine does not alter TBW in healthy men. To date, no studies have investigated the effects of moderate coffee consumption on TBW in caffeine-habituated adults using the doubly labelled water dilution technique.

It is estimated that 1.6 billion cups of coffee are consumed worldwide every day [20], thus it is of interest to know whether coffee contributes to daily fluid requirement, or whether it causes low-level chronic dehydration. In the present study, our aim was to directly compare the effects of a moderate intake of coffee in caffeine-habituated adults against equal amounts of water across a wide range of hydration markers, including the gold standard TBW measure.

## Methods

### Ethics Statement

All participants were informed of the purposes of the study and the risks associated with the procedures. Written informed consent was obtained from all participants before the study commenced. The study was approved by the Science, Technology, Engineering and Mathematics (STEM) Ethical Review Committee at the University of Birmingham, UK.

### Participants

Fifty-two healthy non-smoking males aged 18–46 y were accepted to participate in the study following the screening of over 100 volunteers. Inclusion criteria required participants to be weight stable, pass a general health questionnaire, be free from medications containing caffeine or those that might influence weight or fluid-electrolyte balance, live and work in an environment of ambient temperature with no significant temperature or humidity fluctuations, consume a diet with no extreme food, beverage or dietary supplement intakes and be free from chronic illnesses. Females were excluded from the study due to possible disruptions to fluid balance by the menstrual cycle. Participants were moderate coffee drinkers consuming 3–6 cups per day (300–600 mg/day caffeine) assessed by a 3-day weighed food diary. Two participants could not complete the study due to individual circumstances preventing them from visiting the laboratory.

### Study Design

**Figure 1:**
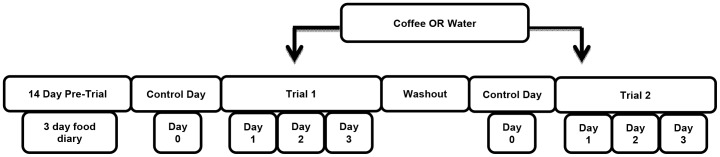
Overview of study design.

Participants reported to the School of Sport and Exercise Sciences, Human Performance Laboratory at the University of Birmingham on two occasions prior to testing. Trials ran from Tuesdays to Fridays during the months of February to December. On their first visit, participants were instructed how to complete a 3-day weighed diet diary and were provided with a set of digital scales (Electronic Scales; Salter Arc) to weigh their food and fluid intake accurately to 0.1 g. On their second visit, participants returned their completed diet diary and baseline body weight was recorded. Each participant completed two treatments each lasting four consecutive days. Each trial was separated by a 10 day wash out period during which time participants were instructed to consume their normal diet and daily caffeine intake. See figure 1 for a schematic overview.

### Treatment

Each trial was undertaken in a counter balanced cross-over design and participants were randomly allocated to a treatment group. The coffee trial (C) involved participants consuming four mugs (200 mL) of black coffee per day (Nescafé Original) equating to a caffeine intake of 4 mg/kg BM. The water control trial (W) involved participants consuming four mugs of water (200 mL) per day. Participants were required to abstain from alcohol and all physical activity 24 h prior to and for the duration of each trial, with the exception of walking for transport.

Diet was controlled and provided to participants throughout each testing period, including the control days. The same diet was replicated and provided for each participant's second trial. Food and fluid intake recorded in the 3-day weighed food diary was analysed for macronutrients, sodium and potassium and food fluid content using nutrition analysis software (Nutritionist Pro, Axxya Systems). Diets were designed and prescribed on an individual basis to replicate mean energy and fluid intakes from the food diary. Diets were a standard weight-maintaining composition of 50%:35%:15% for carbohydrate, fat and protein respectively. A compliance booklet was completed by participants on the morning of each trial day to ensure all food and beverages from the previous day were consumed at the correct times and that no unplanned exertions, fluid losses or nutritional variations had occurred. Participants were instructed to complete the Bristol Stool Chart each morning for indications of disruption to fluid balance [21]. On the morning of each trial, participants reported to the laboratory at a standardised time between 07:00–09:00. Participants were ≥10 h fasted and had not consumed any fluids since 21:30 the previous evening. Participants produced a first morning void (MV) (trial days 1–3) and a separate 24 h urine collection (trial days 2–3). Nude body weight was recorded and a venous blood sample collected. Meals and snacks were consumed at standardised times (±30 min); breakfast at 07:30–09:00 (immediately post testing), morning snack at 10:30, lunch at 13:30, afternoon snack at 16:30 and evening meal at 19:30.

### Beverages

As there is no clear consensus on how much fluid an individual should consume, yet fluid intake was required to be standardised for each participant, daily fluid intake was calculated based on mean individual fluid intakes recorded over the 3-day diet dairy. Daily fluid intake was provided with bottled water and divided into six equal bottles, measured to the nearest gram using digital scales. Participants consumed water at pre-determined time points; between 07:30–09:00 (post testing), 10:30, 13:30, 16:30, 19:30 and 21:30. Test beverages were consumed at predetermined time points; immediately after testing (07:30–09:00), 10:30, 13:30 and 16:30. Each participant was provided with a mug marked at 200 mL. During trial C, participants received four pre-weighed containers of Nescafé Original coffee to provide 4 mg/kg BM of caffeine per day (2.3±0.4 g Nescafé Original per cup) and were instructed to make the beverage with boiled tap water to the 200 mL marker in the mug provided. During trial W, participants were instructed to consume 200 mL tap water in the same mug. Total test beverage intake was 800 mL/day.

## Measures and Analysis

### Blood

Fasted blood samples (approximately 15 mL) were drawn from a superficial vein (21G Venisystem short butterfly, NU Care), typically from the median cubital vein by a trained phlebotomist following a 5 min resting period by participants in the supine position. Fasted blood samples were collected on the mornings of test days 1–3. Blood for serum and plasma analysis were collected directly into two separate tubes; one with clotting agent for analysis of serum sodium (Na^+^), potassium (K^+^), osmolality, creatinine, blood urea nitrogen (BUN) and deuterium oxide (D_2_O) for total body water (TBW) calculation and one with K_2_EDTA for analysis of haematocrit, total plasma protein (TPP) and caffeine. Haematocrit was analysed using a haematocrit centrifuge (Micro Haematocrit Centrifuge, Hawksley & Sons Ltd.) and ruler (Micro Haematocrit Tube Reader, Hawksley & Sons Ltd.). All other samples were centrifuged at 3500 RPM for 15 min at 4°C. Plasma and serum were frozen at −20°C for later analysis. All samples were analysed in duplicate, with the exception of D_2_O. Serum osmolality was determined via freeze point depression via the Advanced Osmometer Model 765 (Advanced Instruments Inc Norwood MA). TPP, serum creatinine and BUN were analysed using the iLab 650 (Instrumentation Laboratory, UK). Plasma caffeine was analysed for participant compliance using a reversed High Performance Liquid Chromatography (HPLC) – UV method, following the protocol described elsewhere [22] (City Hospital, Sandwell and West Birmingham Hospitals NHS Trust). Na^+^ and K^+^ were analysed using Ion Specific Electrodes on the iLab 600 (Midland Pathology Services). Samples were loaded into cuvettes and place into the iLab without pre-preparation and analysed automatically.

### Total Body Water and Body Mass

Nude body mass (BM) was recorded each morning (trial days 1–3) following the participants' first morning void. Participants were fasted and had not consumed any water since the previous evening.

Labelled isotope D_2_O, was provided for each participant to drink to enable the calculation of TBW (99.9 atom % D, Aldrich Chemistry, Sigma-Aldrich). TBW was calculated on days 1 and 3 of each trial to ensure participants began the study in a state of euhydration and to assess any disruptions to fluid balance over the duration of the each trial.

Participants were provided with 0.1 g/kg BM D_2_O, measured to the nearest 0.001 g on day 0 (control day) and trial day 2. Participants were instructed to consume the D_2_O between 20:30–21:30 with their evening water allowance. No additional fluids were permitted following D_2_O ingestion until after the fasted blood sample was collected the next morning (trial days 1 and 3) approximately 10–12 h later. Participants were instructed to continue to collect all urine losses. An additional blood sample was taken on the evening of day 2 (between 17:00–18:00) prior to the consumption of their second dose of D_2_O to establish new blood deuterium enrichment baseline.

Serum D_2_O enrichment was analysed using the Gas-Bench II (Thermo Electron, Bremen, Germany) – isotope mass spectrometry (Finnigan, Delta XP, Bremen, Germany) following the protocol described elsewhere [23]. Briefly, 200 µL of plasma was added to a vacutainer (Labco, High Wycombe, England) with a platinum catalyst (Thermo Electron, Bremen, Germany). The vacutainer was flushed by an automated autosampler-assisted flushing procedure, using 2%H_2_ in Helium gas for 5 min. Following this, a 40 min equilibrium period occurred whereby the hydrogen isotopes in the aqueous solution exchanged with hydrogen ions in the headspace. A sample of the headspace gas was then injected into the Isotope Ratio Mass Spectrometer (IRMS) (Thermo Electron, Bremen, Germany). A mean of the four middle measurements was taken as the measure for each sample. The isotopic enrichment was expressed as δ^0^_/00_ against the international water standard Vienna Standard Mean Ocean Water (V-SMOW). The coefficient of variation of the measurement was 0.027%. Results of the isotope ratio analysis were reported relative to the working reference gas versus V-SMOW and as atom percentage excess (APE).

The delta between sample and reference gas is defined as: *Delta D = [(Ratio of Sample - Ratio of Reference)/(Ratio of Reference)]×1000*. The delta deuterium values for the pre-dose (δpre) and post-dose samples (δpos) were determined. The deuterium dose was diluted with tap water. The deuterium content of tap water (δtap) and the dose (δdose) was measured. TBW in moles could then be calculated from the dilution of the heavy water isotope using the equation: *TBW (moles) = A/(18.02a)×[δdose* - δtap/*(δpost−δpre)*]. A is the amount of dose (g) administered to participants and a is amount of dose (g) diluted for analysis. To convert TBW to kilograms, the following equation was applied: *TBW (kg) = TBW (moles)×18.02/1000 g*. It is known that some deuterium binds to acidic amino acids of proteins or other non-exchangeable sites and it has been experimentally determined that deuterium oxide overestimates TBW by 4% [24]. Therefore, to correct for the non-exchange of deuterium in the body, the TBW measurement was divided by 1.04.

### Urine Analysis

A total of five urine samples were collected during each trial; two 24 h collections (24 h) and three morning voids (V). V was collected separately each day and analysed for urine specific gravity (USG) using a hand held refractometer (Pocket Refractometer, PAL-105. Atago, Japan) and volume using digital scales (measured to 0.01 g) (Sartorius, AG Germany), applying the formula to correct for USG (*V = (M _bottle plus urine collection_−M _empty bottle_)/(USG×ρ_H2O_)*: V is volume (mL), M are masses (g) and *ρ_H2O_* = 1 g/mL is density of water).

Twenty four hour urine collections were analysed for USG and total volume using the methodology outlined above. In addition, 24 h collections were further analysed for osmolality via freeze point depression using the Advanced Osmometer Model 765 (Advanced Instruments Inc. Norwood MA), creatinine on the iLab 650 (Instrumentation Laboratory UK) and sodium (Na^+^) and potassium (K^+^) on the iLab 600 (Midlands Pathology Services Ltd.). The MV volume was added to the 24 h collection to give total 24 h volume data (V24). Following the initial measures of urine volume and USG, urine samples were stored at −20°C for later analysis.

The numbers of urine samples reported throughout the paper are: Volume (*n = 50*), USG (*n = 50*), urine osmolality (*n = 46*), urine creatinine (*n = 48*), urine Na^+^ (*n = 46*) and urine K^+^ (*n = 42*). The numbers of blood samples reported throughout the paper are: haematocrit (*n = 48*), serum osmolality (*n = 49*), total plasma protein (*n = 48*), serum creatinine (*n = 46*), serum sodium (*n = 45*) and serum potassium (*n = 45*). Sample sizes less than 50 are the result of missing samples or technical error.

### Statistical Analysis

All data were analysed using statistical software (SPSS. 18 for Windows). Two-way repeated measures ANOVA with pairwise comparisons post hoc were applied to each data set to look for significant main effects. Delta values were calculated and Student's t-tests were used to analyse changes overtime in each condition. The level of significance was set at p<0.05.

## Results

### Participant Characteristics

Fifty of the fifty two participants recruited for this study fully completed the two trials. The characteristics of the study population are presented in table 2.

**Table 2:** Participant characteristics and pre-trial dietary intakes.

Characteristic	Mean ± SD	Range
Age (y)	28.1±7.3	18–46
Height (cm)	181.1±6.3	169.0–192.0
Weight (kg)	77.0±12.1	51.1–133.6
Total Calorific Intake (Kcal)	2400±464	1402–3398
Dietary carbohydrate (%)	53.5±7.0	33.2–72.4
Dietary Protein (%)	16.4±4.8	10.3–35.3
Dietary Fat (%)	29.8±5.9	14.1–43.0
Total Water Intake	2081±842	1083–3614
Total Coffee Intake (mL)	979±301	625–1522

### Dietary Intakes

The habitual caffeine intake questionnaire and three day weighed diet diaries ensured participants were habitual moderate coffee drinkers with estimated mean intakes of between 300–600 mg caffeine per day from coffee. Any participant whose caffeine intake from coffee fell outside of this range was not included in the study.

During trial C, participants consumed 4 mg/kg day caffeine provided in the form of Nescafé Original, divided into four equal servings of 200 mL. During trial W, participants consumed 200 mL tap water on four occasions each day. Total test beverage intake was 800 mL/day during both conditions. Mean caffeine consumption during trial C was 308 mg, and ranged from 204.4–453.0 mg caffeine.

Compliance booklets suggest that all participants consumed the foods and fluids that they were provided with. In addition, no participants partook in any physical activity, with the exception of walking for transport, 24 h prior to and throughout the duration of each trial. The Bristol Stool Chart monitored any unusual faecal losses. No participants reported unexpected fluid losses from diarrhoea or vomiting during either trial.

Serum caffeine was measured on day two of each trial to check for participant compliance. Results of caffeine analysis, performed by high performance liquid chromatography showed that, as expected, serum caffeine was significantly higher in the coffee trial than the water trial (p<0.01). These findings support participant compliance to the diet and test beverage during each trial.

Dietary macronutrient intake was calculated individually for each participant, based on energy from their food diary. Mean energy intake during the trials was 2425±413 Kcal. Mean water consumption during the trials was 1953±642 mL.

### Body Mass Variables

**Figure 2:**
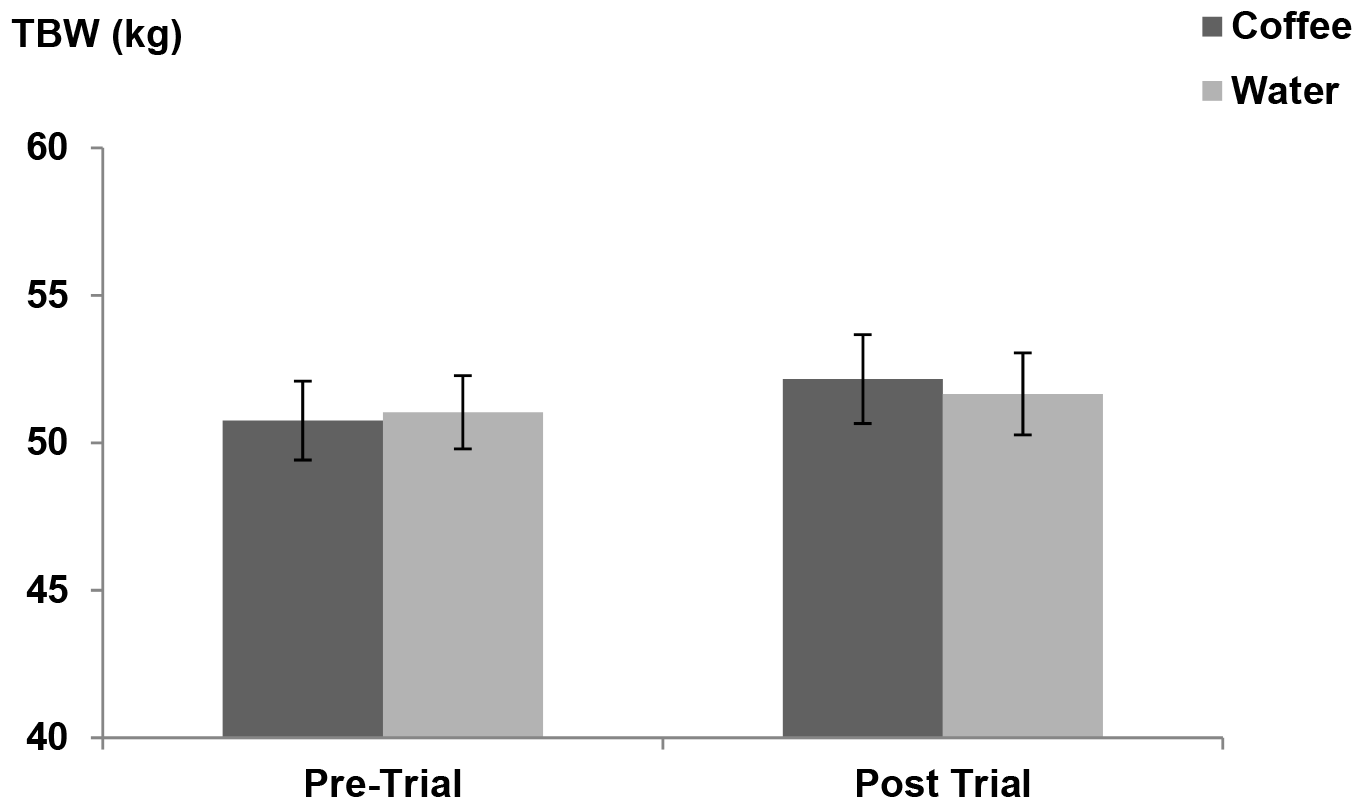
Mean total body water estimates from Day 1–Day 3. *n* = 25.

Figure 2 illustrates the results of TBW estimates, based on deuterium oxide analysis performed by gas chromatography mass spectrometry. Fluctuations in TBW were not significantly different between the two conditions (p = 0.90). There were no significant changes in TBW from beginning to end of either trial (p>0.05) suggesting that participants maintained a stable fluid balance throughout the study. This is further confirmed by statistical analysis showing no significant effect of trial day on either condition (p = 0.43).

**Figure 3:**
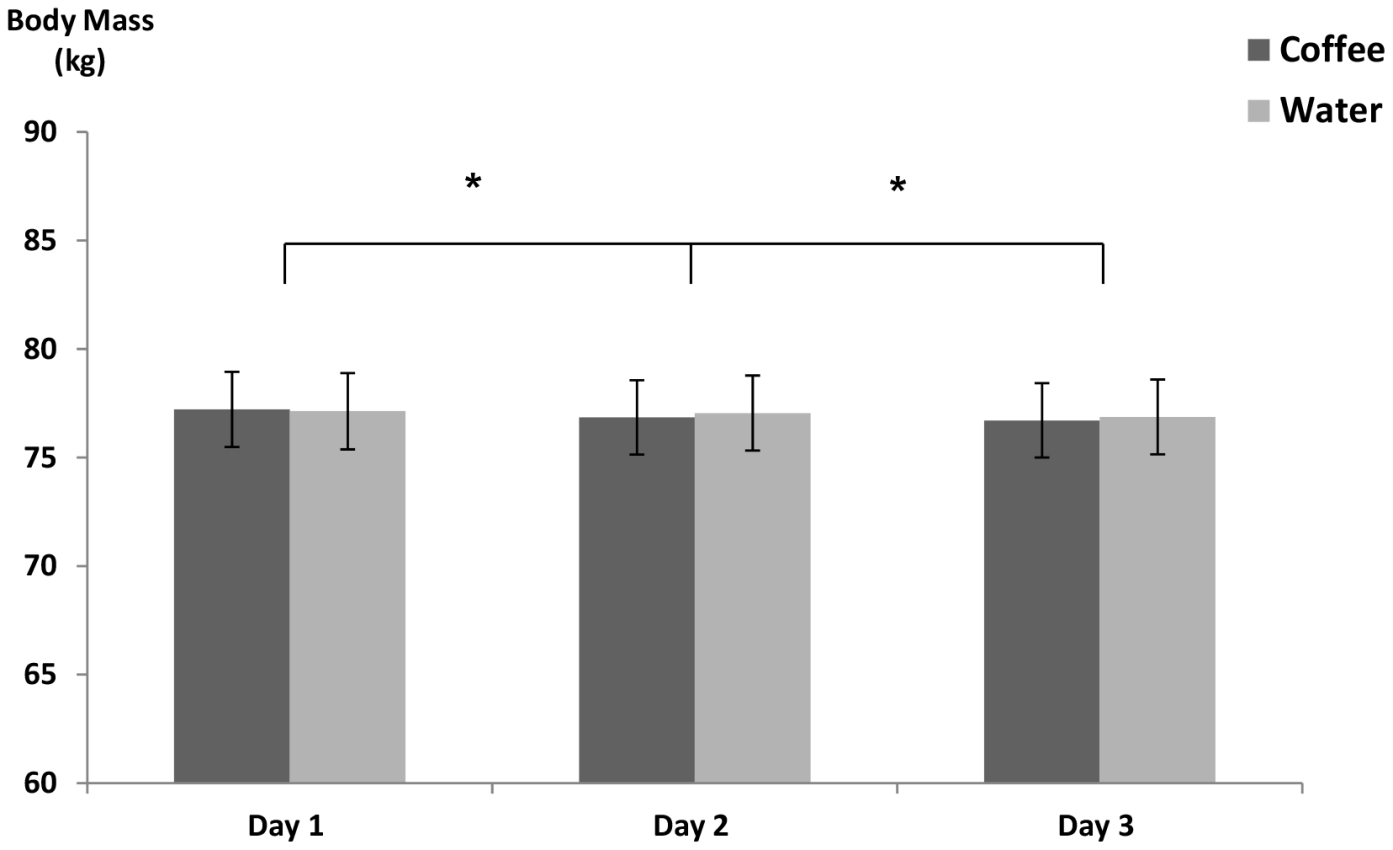
Mean body mass. * Significant difference between days. *n* = 50.

Figure 3 illustrates daily body mass measurements. Mean body mass of participants during experimental trials was 76.97±12.15 kg. Mean body mass did not differ between the two conditions (p = 0.45), however a small but progressive daily fall in BM occurred within both conditions (p<0.05). Mean decrease in BM from day 1 to day 3 across both trials was 0.39±0.5 kg.

### Urinary Markers

Means and standard deviations of 24 h urinary measures recorded on trial days 1 and 2 are presented in table 3. Urine void volume and USG are presented collected on trial days 1–3 are presented in table 4. Twenty four hour urine volume, USG, urine osmolality or urine creatinine did not differ between conditions (p>0.05). Urinary Na^+^ was not different between trials days, however mean Na^+^ excretion was significantly higher on both days in the coffee trial than the water trial (p = 0.02). K^+^ concentration was significantly higher on day 2 in both conditions (p = 0.02), but no between-condition difference was found.

**Table 3:** Twenty four hour urine collection.

Condition/Day	Urine Volume ± SD (mL/24 h)	USG ± SD	Urine Osmolality ± SD (mOsm/kg)	Urine Creatinine ± SD (mg/24 h)	Urine Na excretion ± SD (total mmol/24 h)	Urine K excretion ± SD (total mmol/24 h)
**W1**	2521±744	1.009±0.003	208.2±88.2	624.8±183.4	43.6±15.5	32.3±14.2
**W2**	2335±826	1.009±0.003	202.1±89.8	696.3±288.1	43.4±16.5	36.4±14.0†
**C1**	2593±833	1.009±0.003	217.7±103.2	657.7±243.8	48.7±15.5*	36.6±14.5
**C2**	2226±842	1.009±0.004	243.7±142.7	667.4±271.1	47.0±17.0*	40.3±16.8†

Values are means ± SD.

Coffee significantly higher than water.

Day 2 significantly higher than day 1.

**Table 4:** Urine void volume and USG.

Condition/Day	Urine Void Volume (mL)	Urine Void USG
**W1**	349.2±177.0	1.019±0.005
**W2**	361.9±168.0	1.017±0.006
**W3**	339.8±143.6	1.018±0.006
**C1**	344.6±183.6	1.017±0.005
**C2**	360.1±171.8	1.018±0.005
**C3**	338.3±157.6	1.018±0.005

Values are means ± SD.

Neither urine void volume nor urine void USG were between conditions; p = 0.86 and p = 0.95, respectively (table 4).

### Haematological Measurements

Means and standard deviations of haematological measures recorded on trial days 1, 2 and 3 are presented in table 5. Haematological markers did not differ between conditions across all measures: serum osmolality, haematocrit, total plasma protein, serum sodium, serum potassium (p<0.05). Student's t-test analysis showed no significant differences between conditions in the delta change from day 1 to day 3 for all haematological measures.

**Table 5:** Haematological data collected over each three day trial.

Condition/Day	Haematocrit (%)	Serum Osmolality (mOsm/kg)	TPP (g/L)	Serum Creatinine (mmol/L)	Serum Na (mmol/L)	Serum K (mmol/L)	Blood Urea Nitrogen (mmol/L)
**W1**	43.9±2.0	284±23	73.9±8.1	98.9±11.7	141±4	4.1±0.3	4.6±0.9
**W2**	44.0±2.1	287±11	73.7±6.8	95.9±12.4	141±3	4.2±0.2	4.8±1.0
**W3**	44.0±2.1	285±3	74.2±9.2	95.2±11.2	141±4	4.2±0.2	4.7±0.8
**C1**	43.9±2.3	286±7	73.9±11.0	97.7±13.9	141±3	4.2±0.3	4.7±1.2
**C2**	44.6±2.4	288±8	75.8±9.4	97.5±11.6	141±3	4.1±0.2	4.8±1.0
**C3**	43.9±2.2	286±3	74.7±9.4	97.1±11.7	141±3	4.2±0.2	4.9±1.3

Values are means ± SD.

Renal function was normal throughout each trial as assessed by urine creatinine (Table 3), serum creatinine and BUN (Table 5). Neither urine or serum creatinine differed between conditions or time points (p>0.05).

## Discussion

Individual daily fluid requirements, intakes and beverage preferences vary extensively within and across populations. Despite the lack of a consensus for how much fluid an individual should consume and the effects of various beverages on fluid balance, there are widespread guidelines for optimal hydration that are considered common knowledge [13]. Healthy adults are often advised to avoid caffeinated beverages due to the potential negative impact they may have on hydration status [7]. These opinions and advisories are based upon a relatively small collection of caffeine studies that have been publicised in both scientific and lay literature and in the media over the past few decades [10], [17], [25]. Interestingly however, there is a lack of data that has specifically investigated moderate doses of coffee in free living healthy adults. Thus, the question of whether moderate coffee consumption can contribute to daily fluid requirement remains unanswered.

To our knowledge, this is the first study to directly compare the chronic effects of coffee ingestion with water against a wide range of hydration assessment techniques. We hypothesised that when ingested in moderation; coffee would contribute to daily fluid requirement and would not result in progressive dehydration over the course of 72 h. Our data shows no significant differences in the hydrating properties of coffee or water across a wide range of hydration assessment indices. No significant differences were observed between conditions in any of the haematological markers. No differences in blood urea nitrogen or serum creatinine suggest renal function was normal throughout both trials. Analysis of urinary data showed no significant differences between conditions in 24 h urine volume, urine void volume, USG or urine osmolality. Small daily fluctuations in TBW were observed during both trials; however this did not reach significance in either condition. A very recent study investigated the effects of caffeine provided in capsules (5 mg/kg/day) on the TBW of 30 male participants classified as ‘low-caffeine users’ (<100 mg/day) [19]. No differences in TBW were observed between the caffeine and placebo control group. Our data confirms the author's conclusions that a moderate consumption of caffeine does not disrupt TBW.

Urinary sodium was significantly higher in the coffee trial than the water trial on both days. The increased sodium excretion in the coffee trial falls in line with previous studies that have observed that both theophylline and caffeine enhance sodium excretion at the proximal and distal renal tubules [26]–[28]. The increase in sodium excretion is due to methyxanthine-induced natriuresis caused by inhibition of salt transport along the proximal convoluted tubule [29]. While sodium excretion is an important determinant of urine production, it is not the only driver of urine volume. Some of the water in urine is derived from ‘osmotically free’ water [30], particularly when producing a relatively large volume of dilute urine as in the current study. Baseline urinary potassium was significantly elevated on day one of the coffee trial compared to day one of the water trial. The potassium content in a cup of instant coffee (200 mL and ∼2 g coffee) is approximately 80 mg [30], thus participants consumed ∼320 mg additional potassium during the coffee trial than during the water trial. No differences were observed between the conditions on day two, which may suggest adaptive renal handling.

Interestingly, although no changes were observed in TBW, the data showed a small fall in body mass of 190±120 g/day in both conditions (0.2% BM). Clinical dehydration is reported to be a body mass loss of between 1–3%, therefore whilst the 0.2% BM decrease observed in this study did reach significance; participants were not near the level of clinical dehydration. These findings are similar to the results of Grandjean et al who assessed hydration status in 18 healthy adults consuming a mean caffeine intake of between 1.40–3.13 mg/kg BM [13]. The authors reported a mean loss of 0.30% 0.39 BM across all test conditions. Authors suggested that the loss was due to normal divergence or that their method of determining treatment volumes caused a small level of dehydration to occur. One strength of our study was that the individual fluid intake during the trials was based on three-day diet diaries instead of a fixed volume for everyone. Furthermore, if participants felt they were not allocated a sufficient volume of water at any point during the first trial or indeed if they had too much water, they were permitted to return to the laboratory to have their fluid allocation amended. The adjusted fluid intake was recorded and repeated during the second trial. The small losses in body mass observed in this study are likely to be multifactorial. As suggested by Grandjean et al [13], it is also possible that part of the mass loss observed in this study was due to natural divergence or that participants were not provided with sufficient water during the trials. The urinary data in the current study shows participants were producing relatively large amounts of dilute urine (urine osmolality<serum osmolality), which suggests dehydration is unlikely to be the cause of the fall in body mass. One other possible cause of the body mass loss could be due to unmeasured faecal losses. Participants completed a compliance booklet each day which included questions regarding faecal losses and included the validated Bristol Stool Chart. Data collected from this booklet would highlight any unusual stool production, but as samples were not weighed it is not possible to know how much volume was excreted. No unusual faecal losses were reported by any of the participants.

This study is limited by the nature of its design. To achieve optimal results, a metabolic ward would have provided the most control of the environment and of the participants; however in an attempt to understand the effects of coffee consumption in a ‘free-living’ setting, some control will always be lost. Furthermore, it may have been beneficial to continue the 24 h urine collection on the third day however this was not possible due to time constraints and demands on the participants. It may have been interesting to include a decaffeinated coffee condition as this would have identified any differences specifically caused by caffeine in coffee and not any of the other bioactive components, however as we found minimal differences between coffee and water we believe that it is unlikely that we would have found any significant differences if we had included a decaffeinated coffee condition.

With acknowledgement of the study's limitations, results suggest that coffee did not result in dehydration when provided in a moderate dose of 4 mg/kg BW caffeine in four cups per day. Thus, these data suggest that coffee, when consumed in moderation by caffeine habituated males contributes to daily fluid requirement and does not pose a detrimental effect to fluid balance. The advice provided in the public health domain regarding coffee intake and hydration status should therefore be updated to reflect these findings.
